# Situated psychology as triangulated. Understanding psychological processes as relations between subjectivity, intersubjectivity and objectivity

**DOI:** 10.1007/s12124-025-09916-5

**Published:** 2025-05-26

**Authors:** Bo Allesøe Christensen

**Affiliations:** Aalborg University, Aalborg, Denmark

**Keywords:** Subjective, Intersubjective, Objective, Triangulation, Michael Tomasello, Donald Davidson

## Abstract

This article will attempt to present a framework for understanding situated psychology as involving a dynamic relationship between the subjective, intersubjective, and the objective. Explorations of this relationship within psychology have often highlighted one side at the expense of the others. Here it will be argued, theoretically, that the three sides constitute a dynamic and equal relation working as a background for any psychologically relevant situation, and it is only against this background it makes sense to highlight *one* of the sides. The argument will proceed in three steps. First, I will argue why this notion of triangulation is relevant and furthermore present some reservations on how to understand the triangulation when connected to the situated character of psychology. Indicating that triangulation has a situated character will be argued for using Michael Tomasello's description of children's cognitive development. The point is that there are rudiments very early on indicating psychological situations involving degrees of subjective, intersubjective, and objective elements which are part of human general pscyholog. This will, in the final step, pave the way for a theoretical sketch, with the help of the American philosopher Donald Davidson, of how the three elements are all at play when we try to understand the psychologically relevant in a given situation.

## Introduction

How can we understand situated psychology in a general way? Three possibilities present themselves immediately. First, as an attempt to historically understand psychology as a situated discipline, where concepts, methods, and theories emerge and develop based on historical circumstances (see the article by Birk, Mathiesen, Kirkegaard, and Christensen in this issue). Second, as a question of what the current situation in psychology is, i.e., what themes characterize it, what shifts are occurring between its various branches, and how it is institutionally anchored. Third, as a way of trying to understand psychological processes as situated, asking what this could mean or imply in a general sense. It is this third part I will delve into here.^1^

Recent studies of human psychological development like Tomasello (2009), Hobson (2004), Rakoczy (2008), Zittoun et al, (2007), Martin (2007) or Christensen (2023a) all indicate the importance of the context and the situatedness for the development of psychological capabilities. A situatedness consisting of interrelations, sometimes of equal footing and sometimes not, between subjectivity, intersubjectivity, and objectivity, i.e. not necessarily being reducible to any of the others but often assumed or presented as. If this is the case, then what could possibly be some general philosophical psychological implications of this?

Point of departure will be understanding psychological processes as situated through Tomasello’s way of describing the cultural origins of human cognition. Tomasello’s many studies have provided a very nuanced way of describing the complex development of cognition and its situated character. To draw out some general philosophical psychological implications, I will interpret the American philosopher Donald Davidson’s arguments for the interrelatedness of subjectivity, intersubjectivity and objectivity. The aim of the interpretation is not to develop a new theory, but more of a reminder of understanding this in a situated way, i.e. not reifying or abstracting any sense of either subjectivity, intersubjectivity or objectivity from the practical situations where these are taken as relevant. Hence, the interpretation is more focused on bringing out the situated character and dynamic interrelations between the three parts than Davidson or Tomasello would have emphasized. Emphasis is also more on the Wittgensteinian than other backgrounds to Davison’s and Tomasello’s respective thinking, and in this regard the approach has taken inspiration from Winch (1958) and Cavell (1979).

There are mutual benefits of bringing Tomasello and Davidson together. First, Tomasello mentions Davidson in his newest book (Tomasello, 2021, 45) but does not elaborate. He only registers, that to understand the distinction between subjective and objective, an individual must triangulate on a shared situation with another individual. “…we both see X, but you see it this way, and I see it that way. That is, the participants must come to understand that the two of us are sharing attention to one and the same thing, but at the same time we each have our own perspective on it”. Davidson’s deeper arguments for triangulation, can therefore help elucidate some of the more general psychological implications. On the other hand, as McDowell (2003, 679) pointed out, Davidson seems to presuppose triangulation between subjectivity, intersubjectivity and objectivity as occurring between already full-fledged subjects. Davidson has difficulties in recognizing the importance in acquiring a conception of the world with oneself and others in it “…in ways that pass muster in a group that one is being initiated into.” (ibid.) Tomasello’s description of the triangular nature of cognitive development can serve as a frame for understanding how one is initiated into a conception of a world with oneself, others and things in it.

The primary basis for this article, then, is understanding psychological processes as situated and asking what this could mean in a general sense. I will approach this question by first, inquiring into how subjective, intersubjective and objective aspects emerges as part of human psychological life. Tomasello’s investigations into the cognitive development of humans indicate the existence of rudiments of a situated and triangular character of psychological processes from the beginning. Tomasello's predominantly cultural psychological position has been criticized by'nativists'(primarily followers of Chomsky like Fodor, Hauser, etc.) for not taking the possibility of innate cognitive structures seriously. This debate will not be addressed here. Not because it is not important, but rather because it is not relevant to the article's main point. Second, if we accept Tomasello's understanding of how early cognitive development helps shape the framework for subsequent and mature psychology, how should we understand this in a more general sense? Here, I will include some points taken partly from the American philosopher John Haugeland, and partly from Donald Davidson's considerations on the relationship between the subjective, intersubjective, and the objective. The conclusion we might say, is that the triangulation forms a contexture endogenously enacted by members co- and inter-acting, and which is made available to us through their conceptually meaningfull practices. The article's aim is therefore primarily general psychological, discussing and tentative, but will also try to include brief concrete examples.

## Tomasello: the Early Beginnings of Triangulation

The American developmental psychologist Tomasello has long contributed to understanding human cognitive development and language acquisition. The focus here will be on his early studies (Carpenter et al., 1998; Tomasello, 1999), which can be said to build on the foundational studies of child development by Vygotsky, Bruner, and Piaget, especially for understanding object permanence and intentional behaviour. The point I want to highlight here is that the work of Tomasello and colleagues indicates that our common sense or everyday understanding of the world, including a basic social-psychological and linguistic understanding of other people, is rudimentarily established at a very early age. This is a complex and detailed area, and the following is therefore kept at an overarching level.^2^

## From Dyadic to Triangular Relations

Tomasello and colleagues have within a series of studies pointed to some crucial points in the cognitive development of children. These studies show that the development of understanding social relations, a basic form of subjectivity, and the objective character of the surroundings are rudimentarily formed very early and internally to each other. Hence, understanding the different relationships between subjective, intersubjective, and objective aspects of our interaction with and being in the world becomes part of our everyday understanding from the outset, thereby shaping the situated character of psychological processes.

Around the age of 9 months, Tomasello claims, there is a shift from a dyadic towards a triadic relationship in the child's relation to the world. However, even as an infant, the child shows signs of recognizing and differentiating people from mere physical objects, thereby expressing a basic social understanding. Infants, for example, engage in proto-conversations with caregivers, i.e., social interactions between, for instance, parent and child, where reciprocal attention is established through looks, touches, and simple verbal expressions that together convey a form of shared understanding. Part of this happens through the infant imitating the movements of parents and others, especially movements made with the head and mouth. According to Tomasello (1999, pp. 59–60), this indicates the beginning of a process where the child identifies with conspecifics. This identification-creating transaction is, along with an emerging understanding of communicative turn-taking, primarily dyadic until the age around 9 months:

“If people are around when they are manipulating objects, they mostly ignore them. If objects are around when they are interacting with people, they mostly ignore them” (1999, p. 62).

In the dyadic period of a child's development it appears that a rudimentary understanding of the social is established through a distinction between objects and (primary caregiving) persons, and by relating to the latter in particular ways. Simultaneously, there is also a basic understanding of the physical environment—gradually established through fundamental motor movements and manipulation of objects. According to Tomasello, this changes to a more triadic relationship that both consolidates and develops the relationships established in the dyadic period.

At around 9 months, the child begins to engage in activities of joint or shared attention, i.e., the child gradually establishes an understanding of people as intentional agents, whose relationships to objects or other people can be followed, influenced, and shared by the child. This is a triadic relationship, as it involves the child's attempt to integrate the interactions with and between objects, people, and events which are foci for the joint attention. According to Tomasello, what happens here is that:

”…infants for the first time begin to flexibly and reliably look where adults are looking (gaze following), to engage with them in relatively extended bouts of social interaction mediated by an object (joint engagement), to use adults as social reference points (social referencing), and to act on objects in the way adults are acting on them (imitative learning).” (1999, p. 62).

Now, Tomasello’s wording can sound as if the child is trying to coordinate the relationship between itself, the care-givers and the surrounding world. But as Cavell (1979, 168ff) has argued understanding the relationship this way seems to presuppose too much on the child’s behalf, i.e. that to be able to coordinate the child already needs a thorough understanding of what things and persons are. Rather, and to quote Cavell:

“In “learning language” you learn not merely what the names of things are, but what a name is; not merely what the form of expression is for expressing a wish, but what expressing a wish is; not merely what the word for “father” is, but what a father is; not merely what the word for “love” is, but what love is. In learning language, you do not merely learn the pronunciation of sounds, and their grammatical orders, but the “forms of life” which make those sounds the words they are, do what they do – e.g. name, call, point, express a wish or affection, indicate a choice or an aversion, etc.” (1979, 177–178).

Given this understanding of the beginning of communication or practices of meaning-making, it makes sense to say that children when able to direct caregivers'attention by using deictic gestures such as pointing to external objects, do this internal to different practices. These deictic gestures then gradually serve declarative and imperative functions from within these practices, i.e., functions that aim to make the caregiver aware of something and to get the caregiver to do something. This establishes kinds of normative relationships between the child, the adult, and the surroundings, which may or may not be fulfilled depending on the situation and circumstances in which the gesture is expressed.^3^ Importantly, the child develops a normative understanding internally to these situated triadic situations, that there can be a difference between what one desires and the fulfillment of that desire, between what one expresses and whether it is correct (eg. the child might express hunger but will learn that now is not the right time because…). In this way, the child encounters an independent material and social reality setting boundaries for what is right and wrong, thereby making mistakes possible. The child's emerging understanding, therefore, involves an experience of being able to influence circumstances, but also an experience of not always succeeding, being wrong, and that forming relationships requires effort. This further indicates that alongside an emerging understanding of people as intentional actors, the child also begins to understand itself as an intentional actor. But an intentionality that entails a complex relationship between agency and patiennce, i.e., both active doing, where the environment reacts according to the child's intention, and an emerging restraint, patience, or perseverance in situations that the child does not control, such as when the environment does not react as expected. This initiates the formation of a personified subjectivity, an identity formation like the processes described by Vygotsky or G.H. Mead.

According to Tomasello, the processual triadic relationships serves as an ontogenetic background for the transmission of the material and socio-cultural reality into which the child is born. This is because the child, through the triadic and collective or shared intentionality, is introduced to and initiated into a materially based intersubjective meaningful reality, consisting of material and symbolic artifacts as part of social practices, which are produced and reproduced by intentional actors within the respective culture (1999, p. 1). For Tomasello, the movement from the dyadic to the triadic is common across all cultures, while the concrete realization of the movement depends on the specific culture (see Christensen, 2023a). Here we have, one might say, a form of family resemblance in the natural history of the human life form.

What Tomasello and colleagues'studies seem to indicate then, is that the development of an understanding of the material environment occurs in a dynamic internal situated relationship involving both an understanding of the social, through shared kinds of intentionality, and becoming a self by learning to express oneself through this sharedness. Again, this is a very rudimentary understanding. No child starting out with a triadic understanding of its surroundings can justify its intentions—this requires an understanding of a language's propositional structure as well as which situations require what kinds of justifications. But the adult can often still understand why the child reacts as it does (is thirsty, hence gestures towards the cup). The child's pointing and the adult's reaction in giving the cup thus mark a triangulation—a shared understanding and turn-taking-like transaction, with the child and the adult each manifesting forms of intentionality towards an external object but internal to the given situation. So, in a very rudimentary form, we have here a situated transactional occurrence, where aspects of subjectivity, intersubjectivity, and objectivity are interrelated and develop through each other, but not frictionless.

It is this connection that I now seek to understand in a philosophical psychological sense: what could a triangular understanding mean for our understanding of humans as"minded beings"? But first, I will address a possible (mis)understanding of the above as a simple form of constructivism.

## Prelude to Davidson: Haugeland's Argument Against Social Cartesianism

Before presenting Davidson's view on triangulation, it is worth noting what might be a theoretical consequence of Tomasello's findings. For many years there has been a turn (perhaps several) towards more constructivist positions within the humanities, particularly how these are tied to different understandings of the intersubjective. This is a turn away from a Cartesian starting point where cognition is primarily seen as something internal to the individual and thus, initially, as separate from the socio-material environment. The constructivist idea, instead, is that people do not form their understanding of themselves and their world in isolation, but in relation to and under the influence of other people and institutional contexts. A classic example here would be Gergen's position (Gergen, 1994).

Gergen argues, in contrast to Descartes'famous argument about doubt, that what is crucial is not that doubt cannot doubt itself (as Descartes claimed), but that doubt is a product of our discursive practices. If we can communicate a doubt about certain things, then we cannot really doubt this communication. Therefore, Gergen reformulates the famous sentence attributed to Descartes, cogito ergo sum—I think, therefore I am, to communicamus ergo sum—I communicate, therefore I am (1999, p. viii). It is through communication that humans establish their being as the individuals they are. Gergen's point is that as communication becomes a foundation for our understanding of being, it also entails a fundamental communicative interdependence between individuals, and thus identity formation and social relationship formation are both shaped and unfolded discursively. On this basis, Gergen challenges and replaces an individualistic understanding of the self as a function of cognitive structures with a narrative concept of the self, where meaning is provided through narratively-based social relations. A consequence of Gergen's relationalism is therefore that the process of understanding ourselves does not occur independently of others but is instead a result of continuous communicative participation in a social community. And, by extension, one must assume the same applies to the understanding of the world, i.e., of both artifacts and other people. Any understanding of these must also be dependent on communication, for only thereby is it possible to meaningfully identify and distinguish between different people and things.

Gergen's overall understanding initially seems very plausible and similar to other theories that emphasize the intersubjective or social as the basis for identity formation, self-understanding, and understanding of the world. In continuation of the discussion of externalism in the article by Birk, Mathiesen, Kierkegaard, and Allesøe (2025), one might ask whether Gergen's position actually is more of an extended internalism. The idea behind his attempt to go beyond Cartesian individualism and internalism by emphasizing the social character of meaning-making might not"…so much demolish the Cartesian barrier as merely shift it'outward'a notch"(Haugeland, 2004, p. 258). The problem is that Gergen replaces an internalism within the individual with another internalism within language that is socio-culturally shared but excludes the factual things and concrete events which are part of the situations alongside communication, as being meaningful in themselves. They can only be made comprehensible within the framework of an already given, socially instituted and sanctioned, communication. They cannot then, serve as the necessary correction for something gone wrong or being mistaken, because they are not meaningful in themselves. The consequence of this is that one replaces a foundation of subjectivism (the Cartesian subject) with an intersubjectivism (a socialized subject), which closely resembles replacing one Cartesianism with another, now just a social Cartesianism.

For Haugeland, the originator of this concept, the solution is that we must return to the start. Without knowing or referring to Tomasello, he also points out that we must understand the human being qua human, as initially related to a social *and* material world, both of which are meaningful in themselves. And as Haugeland asserts:

“That means that individual people, everyday social living (including talking), and the everyday world are first intelligible as a unity – that is, as an integrated whole. Only on the basis of that prior whole can those three respective moments be singled out for even relatively focused attention.” (2004, p. 259).

If we are to understand situations psychologically, as being characterized by aspects of subjectivity in the form of unique individual persons, of intersubjectivity in the form of different social contexts, and of objectivity in the form of institutional and material factual criteria, then we must understand these based on a relationality between them—the integrated whole that Haugeland speaks of. Furthermore, it is a relationality that must be understood dynamically, i.e., as transactional, and Haugeland's concept should therefore rightly be understood as implying a whole that, both in the situation itself and in relation to preceding and future situations, is in the process of being understood as integrated.

Donald Davidson shares the same starting point, and his concept of triangulation will be used as an inspiration to understand the dynamic nature of this movement towards a possible integrating whole. The focus here will be on understanding the interplay that occurs between the parts in the formation of a whole, rather than on what the individual parts themselves consist of.^4^ And it is important in this context to be aware that this integrating whole is not necessarily a harmonious whole. A central idea of Davidson's, as it was with Tomasello above, is that the possibility of error is a significant part of the dynamic that occurs between the subjective, intersubjective, and objective.

## Davidson: the Integration of Subjectivity, Intersubjectivity and Objectivity

Davidson is particularly relevant in this context for two reasons. Firstly, like Tomasello, he emphasizes language acquisition as a crucial starting point, which over time helps establish a person's self- and world-understanding (where the world here is both social and material) (Davidson, 1992, p. 120). Secondly, like Haugeland, he goes beyond potential social Cartesianism by pointing to a particular externalist understanding that combines a perceptual and social approach to the world (Davidson, 2001a). In the following, I will start with two assumptions within Davidson's concept of triangulation, respectively a holistic and an externalist starting point, then present the concept itself, and finally summarize how the concept can generally shed light on situated psychology (as transactional and relational). Triangulation is a concept appearing in the later Davidson's writings, i.e., from the mid-1980 s onwards. It is the result of addressing the question"what does it mean to understand each other?"—which is one leading question of his entire work. The concept of triangulation can be described as first appearing in his early thinking about radical interpretation, influenced by Quine's idea of radical translation. The development can then be seen as a movement away from a narrow elaboration of a theory of meaning towards a broader ontological understanding of how mutual understanding of a world shared is achieved (see Ramberg, 1989, and Malpas, 1992). As a way into Davidson's considerations, we can use Tomasello's understanding as presented above and ask, what are the implications of a person acquiring, using, and understanding a language (without distinguishing here between primary and secondary language acquisition)?

## Holism

The first of these is a form of holism, Concepts and beliefs (i.e., our attitudes and understandings) do not appear individually but, as Davidson (1997, p. 124) says, come in"packages."What allows us to identify a belief or concept as opposed to other concepts or beliefs is precisely that they are related to each other. Take a situation where, for example, one sees a dog chasing a cat up a tree. According to Davidson, it would make no sense to say that I have a concept of a tree, which I then connect with a number of other concepts, namely a cat, a dog, the act of chasing, etc. To know just one of these concepts and have a belief, I must know a number of other concepts (and beliefs) and at the same time know how they are connected and different from each other. I must know that what I see in the given situation is not something I see on a screen or dream. I must also know that dogs and cats are animals that have legs, as opposed to trees that do not have legs but have leaves that fall off during autumn. I must also know that one can climb up trees, fall down, and get hurt. As Davidson (1982, p. 98) claims:

“There is no fixed list of things someone with the concept of tree must believe, but without many general beliefs, there would be no reason to identify a belief as a belief about a tree, much less an oak tree.”

A consequence is that just as one cannot have a single belief without many others, there are no beliefs without intentions and emotions (one wants to get the apple from the tree and therefore moves towards the tree to climb it), and conversely, no intentions (to get the apple) without beliefs and emotions (Davidson, 1997, p. 126). Davidson's concept of holism can be interpreted as a natural consequence of the transition to a triadic relationship in Tomasello. The child learns not only to recognize differences between things but also that there are different beliefs associated with different things within different practices (a ball can be rolled, which some food cannot, but both food and a ball can be passed on to someone), and that one can express intentions (as an intentional agent) and that it is okay to express emotions, whether the intentions are fulfilled or not. The child also learns about the correctness of these beliefs, intentions, emotions, etc., both through forms of confirmation and correction and the subsequent actions based on this propriety in other situations. Furthermore, the child gradually learns during language acquisition to express beliefs and intentions through propositions, and how these can be connected through justifications, thereby establishing forms of inferentiality between beliefs, intentions, etc., and their expressions. For example, the statement"I am hungry"can be both a declaration and a justified desire to satisfy hunger. If the answer to the question"what do you want?"is"chips,"a correction may occur in the form of"no chips, we will have dinner soon."Through socialization and language learning, one gradually learns to understand, justify, and express different relationships between beliefs, intentions, emotions within different situations, etc. “We act intentionally for reasons, and our reasons always include both values and beliefs.” (Davidson, 1997, p. 125) Two additional points concerning holism are important here.

Firstly, to identify beliefs, intentions, emotions, etc., and express these propositionally, one must have concepts. According to Davidson, this is one difference between animals and humans, that humans form judgments in a special way because they have concepts, which animals do not have. Animals can of course distinguish between things and other animals in the environment, but this does not mean that their ability to distinguish is conceptual. Having a concept only makes sense if one has an idea of"…misapplying the concept…"(Davidson, 1997, p. 124), such as inferring from being hungry that one is (wrongly) entitled to get chips as in the example above. Having concepts and being able to express them opens a space for mistakes that do not exist by animals. A dog can distinguish a cat from a mouse but not understand the cat as a cat or the mouse as a mouse and thereby understand itself as confusing a mouse and a rat. The dog does not understand what follows from a cat being a cat, i.e., the relations the concept of a cat has to other concepts, values, beliefs, but in contrast to other concepts, values, etc. As socialization and language learning form a background for understanding different situations, this background allows us to understand when concepts are used incorrectly, intentions are misunderstood, and feelings can be misplaced. For Davidson, this is important because here objectivity shows itself internally to the different situations in which mistakes occur. This comes with a normative obligation to correct the error in question as well, by accepting that the authority lies with what we are mistaken about, similar to Haugeland above.

Secondly, when beliefs, concepts, values, etc., come in"packages,"they tend to support and give content to each other:

“As a result, unless one’s beliefs are roughly consistent with each other, there is no identifying the content of beliefs. A degree of rationality or consistency is therefore a condition for having beliefs.” (Davidson, 1997, p. 124).

Implied is not that all persons are perfect rational beings. One can certainly be committed to two incompatible beliefs (like demanding chips even though dinner is coming up soon)–this is often the case–the inconsistency lies more in claiming that one is entitled to both. And even such an inconsistency is only possible against the background of a fundamental"…space of reasons; inconsistencies are perturbations of rationality, not mere absence of rationality."(Davidson, 1997, p. 125). According to Davidson, the same point applies to how we understand others, namely as expressing a certain consistency between their feelings, beliefs, and values in relation to what they say and do. This can, in many ways, be related to different roles or positions, as described in sociology of Goffman or the social psychology of Harré, and associated with different forms of consistency in our understanding of these roles or positions.

Davidson connects this with what he calls *the principle of charity*, i.e. interacting with people we understand these in a coherent sense,"…discover a degree of logical consistency in the thought of the speaker."(Davidson, 1991, p. 211). Just as it is impossible to have a belief without several others, it is a condition for our understanding of each other that we attribute a coherence of mostly true concepts and beliefs to each other. Again, this can be understood as a part of the human development presented by Tomasello. The socio-cultural contexts forming the background for children's upbringing sets an internal limit to the diversity of beliefs, emotions etc. we attribute to each other in specific situations. However, Davidson also claims this should not be understood as everyone (within a culture or family) are sharing the same perspective on the world or being fundamentally in agreement initially. Rather, understanding each other is something we are always moving towards, and the principle of charity is a condition for this. As one of Davidson's students claims about the principle, it"…provides an initial specification of beliefs that is intended to enable the interpretive process rather than complete it."(Malpas, 2011, p. 262).

This also applies to a related principle, namely the principle of correspondence, which"…prompts the interpreter to take the speaker to be responding to the same features of the world that he (the interpreter) would be responding to under similar circumstances."(Davidson, 1991, p. 211). We are approaching the second assumption here, as these features of the world are understood in an externalist sense and connect the two speakers in their relation to each other.

## Externalism

Externalism can generally be described as the idea that our beliefs, values, etc., all that we express through our conceptuality, are conditioned by the socio-material context we are embedded in. For Davidson, externalism is"…a view that makes the connection between thought and world intrinsic rather than extrinsic – a connection not inferred, constructed, or discovered, but rather there from the start."(Davidson, 2001b, p. 2).^5^ Like Haugeland and Tomasello's respective points, relations to the world are understood as meaningful by virtue of an internal connections to the situations or contexts in which these relations are formed. And it is this internal connectedness that is a condition, as an integrated whole, for even talking about relations (to oneself, to others, and to the surroundings) as separate. For Davidson (e.g., 1990; 2001a), there is, however, a particular connection between a perceptual and social externalism that applies.

In Davidson (2001b, p. 2), he presents his own view more closely by pointing out the challenges of these two:

“…*social externalism*, which maintains that the contents of our thoughts depend, in one way or another, on interaction with other thinkers; and *perceptual externalism*, which holds that there is a necessary connection between the contents of certain thoughts and the features of the world that make them true.”

For Davidson, the problem with both is they cannot separately account meaningfully for the objectivity of our beliefs. The perceptual because it assumes that a person's (the subject's) perception is independent of social conditions, and the social because it assumes intersubjectivity by itself is enough to establish an objectivity independent of the reality in which the intersubjective is embedded and directed towards. Let's consider both separately.

For social externalism, in Davidson’s view, the correctness of a sentence depends on the possibility that other speakers will utter the same sentence in a similar situation (e.g., speaker 1: there is a cat; speaker 2 standing right next to speaker 1: yes, that is a cat). The correctness or objectivity is here defined or established by the intersubjectivity or what the two speakers share, experience, or encounter in the given situation. Often, this is understood as a form of norm conformity, as"…correctness is defined as going with the crowd."(Davidson, 2001b, p. 3).^6^

But is two or more people doing or saying something in the vicinity of the same thing enough to claim an objectivity, a correctness or incorrectness of their corresponding sentences or actions? Not according to Davidson, because merely following socially established norms or standards of correct behaviour, including linguistic behaviour, cannot *by itself* establish the correctness or incorrectness of what is said or done. This resembles Haugeland's concept of social Cartesianism, where even cases with"…divergence, even when combined with sanctions to encourage conformity, do not introduce the sort of norm needed to explain meaning or conceptualization."(Davidson, 2001b, p. 4), and thus the objectivity of our'mindedness.'In other words, the social context is necessary but not sufficient to understand objectivity. It is only necessary because it is through engagement and commitment to others, through socialization and dialogue, that the possibility of differences in understanding the same thing can emerge. So, although social conditions are not in themselves enough to understand objectivity or the possibility of error, they create a"…space for something that can be called error: room for error is created by cases in which one individual deviates from a course of action when the crowd does not."(Davidson, 2001b, p. 5). Here, perceptual externalism comes into play. Perceptual externalism can be seen as the complementary element missing in the social externalist understanding, namely how content is linked to conceptuality and thus, how an objective relation to the world is established. Davidson discusses this in the context of how perceptual beliefs are causally established in relation to the world.

In the discussion of perceptual externalism, Davidson argues against simplified empiricist positions that construct our relation to the world as consisting of epistemic intermediaries, such as sense data or impressions. The point is not to assess whether these things exist or not, but whether they are basic in relating to the world. If they are considered as such, according to Davidson, it quickly results in a form of subjectivism (in the sense of solipsism) because one can never know anything about what causes these epistemic intermediaries, since it is only these intermediaries one has access to.^7^ The argument against epistemic intermediaries means that my perception of seeing a red apple on a tree must instead consist of a kind of causal relation between me and the environment in which the tree and apple are located. In Davidson's understanding, reaching out or pointing at the apple would thus be a reaction to my perceptual stimulus of the apple. The tree with the apple provides an affordance for me to do something, e.g., eat or throw the apple. In both cases, whether I want to eat or throw the apple, the content of these beliefs or intentions must in some way be influenced – in a good externalist sense – by what directly prompts them. It would not make sense to say that I form the belief that I can eat the apple, or form the intention to throw it, if I am not perceptually convinced that the apple is *actually* there.

But to claim that the world somehow causally conditions our beliefs presents two challenges. Firstly, what is the decisive factor in the causal chain? If we assume a common understanding of how cause-and-effect relationships are established, we will point to the immediate cause – i.e., the apple in the example above. But for Davidson, this is not particularly helpful in understanding what we refer to on the scale between distal and proximal stimuli. Is it the energy or light reflected from the object or the stimulation of nerve endings, or perhaps a third we mean by the immediate cause? This suggests that the content of our perceptual belief is more indeterminate than one would think, because the causal relation, in itself, cannot give meaning to which specific objects or features of the world actually contribute with the given content. Secondly, if we cannot be certain what the actual cause of our belief is, how can we know that we are not making a mistake? Instead: “What must be added in order to give an account of error is something that can count as recognition or awareness, on the part of those who share reactions, of each other’s reactions.” (Davidson, 2001b, p. 5).

For Davidson Shared Reactions Like These.

”…between two people in the presence of stimuli from a shared world contain the kernel of ostensive learning, and it is only in the context of such interactions that we come to grasp the propositional contents of beliefs, desires, intentions, and speech.” (Davidson 198, p. 86).

So, what ensures that we can be certain about our perceptions is that other people interact with the same world, so that people classify and react more or less in the same way: “Without this sharing of reactions to common stimuli, thought and speech would have no particular content – that is, no content at all. It takes two points of view to give a location to the cause of thought, and thus to define its content.” (Davidson, 1991, pp. 212–213).

In other words, a normative understanding is attached to what we perceptually experience by being related internally to shared practices of responding to common stimuli, because otherwise, we “…have no reason to claim that others were responding to the same objects and events (i.e., causes) that we are.” (Davidson, 1990, p. 202). When I ask if you will get the apple for me, and I point to it, this is not merely a causal relation but also an expression of shared attention to the surroundings, manifesting it itself though the words I use and how these gain meaning though the role they play in our practices. I understand, for example, how to use the term"apple"correctly for what I see in my environment, but also that I can ask you to get it, and that you understand what I am referring to. There is also room for error, for example, that I may not be allowed to take the apple, making it wrong to grab it, or that you may think it is a different apple I am pointing to.

Perceptual and social externalism complement each other. It is through interaction with others that the perceptual content of our understanding of the world is often established, and it is by virtue of perceptual externalism that social interactions become more than merely expressions of norm-related conformity. Davidson captures this in an almost poetic way when he asserts that *the possibility of thought comes with company*, namely from both humans and the world around them. This does not override the subjective aspect in the triangulation, but sees it in relation to the objective and the intersubjective. As Davidson expresses it:

“Our thoughts are'inner'and'subjective'in that we know what they are in a way no one else can. But though possession of a thought is necessarily individual, its content, is not. The thoughts we form and entertain are located conceptually in the world we inhabit, and know we inhabit, with others. Even our thoughts about our own mental states occupy the same conceptual space and are located on the same public map.” (1991, p. 218).

The subjective, the intersubjective, and the objective belong together without being reduced to each other (Davidson, 2001a, p. 13). They exist in dynamic relations to each other, but relations not necessarily expressed as harmonious wholes. These are the form of relations that Davidson understands through the notion of triangulation.

## Triangulating

Triangulation thus involves"…a minimum of three elements: two creatures and a world of objects, properties, and events the creatures can discriminate in perception"(Davidson, 2001b, p. 5). This should be understood as dynamic transactions with changing and shifting relations between individuals and the world that surrounds them. Several points can be addressed here. Firstly, the persons (creatures in the quote) interacting through triangular processes are positioned dynamically through the meetings of the relations:

“It is the result of a threefold interaction, an interaction which is twofold from the point of view of each of the two agents: each is interacting simultaneously with the world and the other agent…each creature learns to correlate the reactions of other creatures with changes or objects in the world to which it reacts.” (Davidson, 1997, p. 128).

The possibility for error here is “…the occasional failure of the expectation; the reactions do not correlate” (Davidson, 1997, p. 129). The difference between having a belief, a thought, or intention and what is the case becomes manifest here, and hence also a possible space for correcting any mistakes. In the process of correlating, as referred to in the quote, an additional relation is in play, namely the individual’s relation to themselves. So, the relation to the world and to the other also contains a personalized relation:

“But what individuates that state at the same time makes it accessible to others, for the state is individuated by causal interplay among three elements: the thinker, others with whom he communicates, and an objective they know they share” (Davidson, 1990, p. 204).

I know who you are, not by understanding you as isolated from everything else, but through the relations you have with the world and me. Triangulation therefore consists, ontologically, of the interplay between three elements: the subjective, intersubjectivity, and the objective, or the personal, the social, and the world in which these are embedded.

Secondly, and as a natural consequence of his holism, Davidson argues against reducing these three elements to each other. When beliefs are constituted by the relations existing between them, they cannot be reduced to each other without losing their distinctiveness. The same applies to the three forms of knowledge associated with triangulation: knowledge about oneself, knowledge about the world, and knowledge about other people. For Davidson, this mutual relation is particularly strong because without just one of them, the other forms of knowledge would not be what they claim to be about. Davidson (1991, p. 220) uses a three-legged stool as a picture for this mutual dependency, because all three legs must be there for the stool to stand. He also asserts that “…all three varieties of knowledge are concerned with aspects of the same reality; where they differ is in the mode of access to reality.” (Davidson, 1991, p. 205). As we saw above with Tomasello, it is part of children's development that they form an understanding and knowledge of all three spheres simultaneously. So, to have knowledge of one of these spheres, one must also have knowledge from the other spheres:”…since the basic triangle is a condition of thought, but none (of the other kinds of knowledge) is conceptually prior to the others.” (Davidson, 1998, p. 87).

Obviously, there is a difference between a child involved in early language learning and a competent language user. For the child, there is a socio-cultural initiation into engaging the world though different practices and forms of life that is conceptual and temporally prior; otherwise, there would be no content the socialization and cognitive development of the child. But both are still marked by a relationship between the three elements as an interconnected whole. In Davidson's words:

“Without this sharing of reactions to common stimuli, thought and speech would have no particular content – that is, no content at all. It takes two points of view to give a location to the cause of thought, and thus to define its content.” (Davidson, 1991, pp. 212–213).

Furthermore, it would be impossible to distinguish between what I am convinced of and what is actually the case if I did not triangulate with another person. To correlate our responses, I must be able to differentiate your response from mine, and specifically when I try to understand whether our responses are practically directed towards the same thing or event in our shared environment. Both our responses are individuated through the actions, words, or gestures we use in the (different) practices we share. Our thinking is, as Davidson (1991, p. 218) asserts, subjective"…in that we know what they are in a way no one else can. But though possession of a thought is necessarily individual, its content is not."Therefore, the subjective is not the same as the arbitrary. For even though all my thoughts are mine, they are also conceptually shaped within a social nexus with other people interacting with the environment, thereby providing content to these thoughts and thus establishing their possible correctness^8^:”Intersubjectivity is the root of objectivity, not because what people agree on is necessarily true, but because intersubjectivity depends on interaction with the world.“ (Davidson, 1998, p. 91).

To reiterate, for Davidson the three kinds of knowledge cannot be reduced to each other. Instead, the point is that to understand one of these modes of knowledge, we must simultaneously understand how it is related to and dependent on the other modes.

## Consequences

Davidson's understanding of triangulation has several consequences for a theoretical understanding of a situated psychology. Firstly, the immediate consequence is that psychological phenomena and events are underpinned by forms of triangularity expressed through different conceptual practices. Psychological phenomena are stretched out between and influenced by personal, material, and social conditions or prerequisites initially interconnected but in different ways and in different situations. To understand or study such phenomena as situated, one must try to understand how these triangular aspects are dynamically in play.

Secondly, Davidson's understanding can indicate the limitations of psychological understandings recognizing only parts of the aspects in the triangulation or reduce the complexity of psychological phenomena to only one of these. This could, for example, be a critical psychology, where'the self'or a first-person approach is dominant initially, in contrast to a more experimental psychology that has a more objectifying third-person approach, and an intersubjectively influenced social psychology.^9^ This is not to claim that one cannot investigate one of the parts in the triangular ontology, as different research paradigms do. But it means recognizing, first, that knowledge of the other parts in the triangle is not only to be presupposed but often also necessary to understand a specific study. Second, given the non-reducible character of the different aspects of the triangulation should be accepted, the interpretation of Davidson's understanding here can be seen to suggest investigating and understanding psychological phenomena in an interdisciplinary fashion.

Thirdly and lastly, these considerations point towards understanding psychological phenomena through what could be termed a transactional relationism. It is through people's practical inter- and transactions with each other and the world they are part of, that identifiable relations are created, making the subjective, the intersubjective, and the objective individualized and concretized. These interactions and relations are not necessarily harmonious and symmetrical, because misunderstandings and mistakes are to be understood as a meaningful part of triangulation. This occurs often as interactions in different situations, where the relation between what is meant and what is actually happening, as Davidson puts it, cannot or will not be acknowledged. Triangulation might also help provide a broader perspective on psychopathological phenomena. Tomasello, for example, has pointed out that infantile autism often manifests itself in the transition to a triangular relationship, with the child having difficulties establishing emphatic relations to others. A triangular approach for understanding this would be interdisciplinary investigating the complex relationship between the objective and the intersubjective, the physiological, environmental, and social conditions, as these affect the specific case of autism. In general, a situated psychology must therefore consider all the triangular aspects of psychopathological phenomena as these are manifested though different situations.

## Conclusion

This article has presented an understanding of psychological processes as situated and involving dynamic relations between subjective, intersubjective and objective elements. This situatedness has been described developmentally with the help of Tomasello, and it was indicated that a triangulation of relations between people and the world they are in already had a decisive importance here. An argument was made against understanding triangulation in an overly constructivist sense, as it could imply a form of social Cartesianism, making it difficult to understand the significance of making mistakes as a difference between what one means, says, and does, and what is actually the case. Instead, it was pointed out that triangulation should be understood as a dynamic relation between the subjective, the intersubjective, and the objective. This was described more thoroughly with the help of Donald Davidson's understanding of triangulation as combination a holism of psychological concepts with a social and perceptual externalism.

## Data Availability

No datasets were generated or analysed during the current study.

## Footnotes

1 This article is a based on a paper in Danish that has been significantly revised and adjusted to the present context; see Allesøe (2024)

2 One point I will not address here is the extent to which Tomasello's understanding of children's development is committed to a theory of mind. I will only note that in Tomasello's latest work – with the establishment of what he calls a neo-Vygotskian understanding – he is critical of the individual orientation that a theory of mind might presuppose and highlights the social background in the form of collective or shared intentionality more than in his early work (Tomasello, 2021pp. 84–85).

3 The child can gesture towards a drinking cup or perhaps a window, but also towards the coffee cup or the candle on the table near the child's cup. The adult respectively allows or opposes the child's attempts by marking and articulating what the child can and cannot do—for example,"ah, you are thirsty, here is your cup"or"do you want your cup, here it is". But also"Yes, the light is beautiful, but it hurts if you touch it."Often this is combined with gesticulation and paralinguistic elements.

4 I am thinking here, among other things, that subjectivity can be content-wise linked to an individual's corporeality, as well as the individual as a person, intersubjectivity to socio-cultural institutional contexts but also to a contemporary concept such as intercorporeality, and finally, objectivity can be linked to both natural and social contexts and facts, and associated – as already indicated – with concepts such as niche or Umwelt

5 I take Davidson’s point, perhaps not followed through by him, here to be Wittgensteinian: encountering something not easily understandable from a psychological point of view it is often enough to just remind ourselves of the situated but complex character of psychological processes and how they are articulated through language. We are not necessarily discovering something new or in need of making inferences to establish some new theory. In many cases reminding ourselves of what we already know or can imagine taking place appealing to the intrinsic relation between the different uses of language in different practical situations is enough for a start. Hence, the claim in the introduction that the aim here is not developing a new theory, but a reminder or conceptual clarification of the situated character of psychological processes as involving subjective, intersubjective and objective aspects.

6 Consider this broadly, as instances where power relations can come into play, where one'conforms'to the viewpoint that is most beneficial for oneself (for example, for advantageous or fearful reasons), or social psychologically as conforming to a group.

7 The conclusion of Davidson's argument is very similar to the conclusion of Gibson's argument for direct perception (Gibson, 1986). Gibson says, for example, that perception does not consist of stimuli as discrete inputs, but as stimulation, i.e., more as a form of holistic stimulation of the senses (1986, pp. 57-58). Without being able to argue in depth here, I would suggest that Davidson's argument could be strengthened by linking it to Gibson's considerations around ecological psychology (see also Heft 2001). The reader will probably note some of Gibson’s concepts in the interpretation of Davidson here.

8 A reviewer asked about triangultion as applied to media platforms and concepts like fake news. This is a big issue I wont be able to deal with in depth here. The arguments presented here are obviously directed at communication based on interpersonal relations, but they might also apply to more technological mediated communication like social media platforms. These platforms and the interactions occurring on and through them, can still be characterised as triangular, only each platform provides different technological means or conditions for triangular processes to take place. Following Christensen (2023b) a notion of triangulation might therefore help making explicit the different and similar conditions these platforms implicitly set up for the subjective, intersubjective and objective aspects of interactions to unfold. This could help increasing the critical awareness of how these platforms thereby influence the situated character of the interactions taking place, and, perhaps more importantly, the efforts of dissolving any distinction between what is expressed on the platforms and what is actually the case, as in fake news.

9 It could also be phenomenology about the subjective, cultural and social psychology about the intersubjective, and materiality studies about the objective.
